# Functional Role of Milk Fat Globule-Epidermal Growth Factor VIII in Macrophage-Mediated Inflammatory Responses and Inflammatory/Autoimmune Diseases

**DOI:** 10.1155/2016/5628486

**Published:** 2016-06-27

**Authors:** Young-Su Yi

**Affiliations:** Department of Pharmaceutical Engineering, Cheongju University, Cheongju 28503, Republic of Korea

## Abstract

Inflammation involves a series of complex biological processes mediated by innate immunity for host defense against pathogen infection. Chronic inflammation is considered to be one of the major causes of serious diseases, including a number of autoimmune/inflammatory diseases, cancers, cardiovascular diseases, and neurological diseases. Milk fat globule-epidermal growth factor 8 (MFG-E8) is a secreted protein found in vertebrates and was initially discovered as a critical component of the milk fat globule. Previously, a number of studies have reported that MFG-E8 contributes to various biological functions including the phagocytic removal of damaged and apoptotic cells from tissues, the induction of VEGF-mediated neovascularization, the maintenance of intestinal epithelial homeostasis, and the promotion of mucosal healing. Recently, emerging studies have reported that MFG-E8 plays a role in inflammatory responses and inflammatory/autoimmune diseases. This review describes the characteristics of MFG-E8-mediated signaling pathways, summarizes recent findings supporting the roles of MFG-E8 in inflammatory responses and inflammatory/autoimmune diseases, and discusses MFG-E8 targeting as a potential therapeutic strategy for the development of anti-inflammatory/autoimmune disease drugs.

## 1. Introduction

Inflammation is the first-line of defense protecting our bodies from harmful stimuli, including pathogen invasion, irritants, and apoptotic or damaged cells [1, 2]. The features of inflammation include heat, pain, redness, swelling, the recruitment of various immune cells to the inflammatory tissues, the destruction of tissues, and the dysfunction of organs. Inflammation is categorized as either acute or chronic. Acute inflammation is an immediate immune response at the injured or infected sites, whereas chronic inflammation that develops from continuous inflammatory stimuli involves prolonged immune responses that can last for several weeks to years and is characterized by cycles of active inflammation, tissue injury, and healing. More importantly, chronic inflammation is a major causative factor of a number of inflammatory/autoimmune diseases, including rheumatoid arthritis, systemic lupus erythematosus, asthma, psoriasis, atherosclerosis, diabetes mellitus, and Alzheimer's disease [3–6]. The inflammatory response is a complex biological process that actively involves many types of inflammatory immune cells. Among these immune cells, macrophages, a type of white blood cell that is developed in the myeloid cell lineage, are key players in the inflammatory response. Once macrophages are activated by inflammatory stimuli, they initiate inflammatory responses and produce inflammatory mediators such as nitric oxide (NO), prostaglandin E_2_ (PGE_2_), reactive oxygen/nitrogen species (ROS/RNS), and proinflammatory cytokines such as tumor necrosis factor-alpha (TNF-*α*), interleukin-1 beta (IL-1*β*), and IL-6 through activating intracellular signaling cascades composed of different types of kinases and transcription factors [7–15]. Macrophages are activated and then turn on host defense mechanisms in various ways, such as recognizing pathogen-associated molecular patterns (PAMPs) through pattern recognition receptors (PRRs), phagocytosing pathogens through receptor-mediated endocytosis, and receiving various molecular signals released from other immune cells. Interestingly, recent studies have demonstrated that macrophages are activated and play a crucial role for host defense in inflammatory responses and inflammatory/autoimmune diseases via direct phagocytic engulfment of damaged or apoptotic cells through the formation of macrophage-damaged/apoptotic cell complexes mediated by a “molecular bridge,” such as milk fat globule-epidermal growth factor 8 (MFG-E8).

An unknown cDNA sequence and its corresponding protein were first identified in 1990, and its DNA sequences were revealed in mouse mammary epithelial cells [16]. This gene was named MFG-E8 because it was highly concentrated in milk fat globules and its amino acid sequences were similar to those of both epidermal growth factor (EGF) and blood coagulation factor V/VIII. MFG-E8, also known as lactadherin or secreted epidermal growth factor repeat and discoidin domain-containing protein 1 (SED-1), is a secreted protein that was initially found as a bridging molecule between damaged or apoptotic cells and phagocytic cells such as macrophages through the release of “eat-me signals” that lead to the macrophage-mediated phagocytic clearance of damaged or apoptotic cells [17–19]. Given its role in clearing damaged/apoptotic cells from various tissues, MFG-E8 has been considered to be an essential regulator of the progression of many inflammatory/autoimmune diseases, and a variety of fields, including pharmacology, molecular medicine, and innate immunity, have begun to study its effects in several experimental animal models of inflammatory/autoimmune diseases [20, 21].

In this review, we provide a general introduction to MFG-E8 as a mediator of damaged/apoptotic cell clearance and discuss the roles of MFG-E8 in macrophage-mediated inflammatory responses and inflammatory/autoimmune diseases. Moreover, we further discuss the targeting of MFG-E8 as a potential therapeutic strategy for various inflammatory/autoimmune diseases. The aim of this review is to increase the understanding of the roles of MFG-E8 in macrophage-mediated inflammatory responses in order to provide new insights that can aid in the development of anti-inflammatory drugs for the treatment of macrophage-mediated inflammatory/autoimmune diseases.

## 2. Structure and Functions of MFG-E8

### 2.1. Structure and Posttranslational Modification of MFG-E8

**Figure 1:**
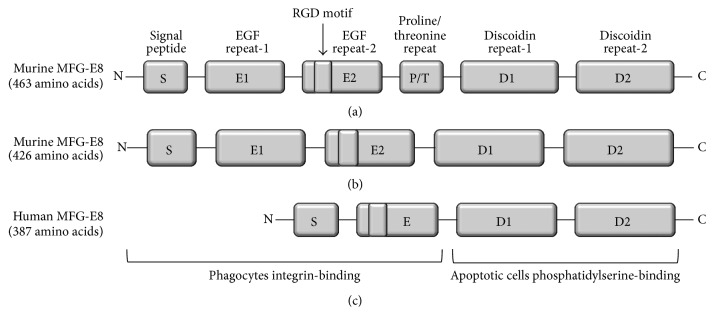
Structures of the (a) long form and (b) short form of murine MFG-E8 and (c) human MFG-E8.

MFG-E8 is a 46 kDa secreted glycoprotein consisting of an N-terminal signal peptide that directs MFG-E8 into the extracellular environment, EGF repeats, and discoidin domains (Figure 1(a)) [19]. The EGF repeats and discoidin domains were named because of their sequence similarities to EGF and blood coagulation factor V/VIII, respectively. Both the EGF repeats and discoidin-like domains have two similar copies (EGF repeat-1 and EGF repeat-2 and discoidin domain-1 and discoidin domain-2, resp.), and these bimotif structures facilitate the recognition and scavenging of apoptotic cells from tissues [19]. The second EGF repeat contains a highly conserved arginine-glycine-aspartate (RGD) motif, which is critical for binding with the *α*_v_ *β*_3_/*α*_v_ *β*_5_-integrin of phagocytic cells. The two discoidin-like domains recognize apoptotic cells through their interaction with anionic phosphatidylserine/phosphatidylethanolamine (PS/PE) (Figure 1(a)) and also bind with collagen through their homologous sequences present in the collagen receptors DDR1 and DDR2, which mediate the turnover of the extracellular matrix [22]. Two isoforms of murine MFG-E8, a long and short form generated by alternative splicing, have been identified [23]. The main difference between these two forms is that the long form contains proline/threonine-rich repeats (P/T) between its EGF repeat-2 and discoidin domain-1, while the short form does not (Figure 1(b)). The structure of human MFG-E8 is simpler than that of murine MFG-E8. Human MFG-E8 has two discoidin domains similar to murine MFG-E8 but only has one EGF repeat (Figure 1(c)). Two isoforms of human MFG-E8 have been reported as splicing variants. Isoform 1 differs from the canonical MFG-E8 because it is missing amino acid sequences from 1 to 75 and isoform 2 is even shorter than isoform 1, missing amino acid sequences 291–342 of the canonical MFG-E8. MFG-E8 is structurally and functionally homologous to DEL-1 (developmental endothelial locus-1), which plays critical role in the phagocytosis of apoptotic cells [24]. DEL-1 binds with PS and integrins on apoptotic cells to induce phagocytic activity.

MFG-E8 is highly glycosylated* in vivo* [23, 51–53]. Although MFG-E8 has been found to be glycosylated on both N- and O-glycosylation sites, O-glycosylation only occurs on the threonine residues in the P/T-rich repeat regions in the long form. The glycosylation sites can easily be modified to facilitate the interaction between apoptotic cells and macrophages, and this interaction delivers signals to the macrophages to consume the apoptotic cells [53]. MFG-E8 is phosphorylated as well. Using the CRISPR/Cas9 genome editing technique, mass spectrometry, and biochemical analysis, MFG-E8 has been identified to be phosphorylated on the serine 42 residue by Fam20C kinase [54].

### 2.2. Expression and Localization of MFG-E8

MFG-E8 is a peripherally secreted glycoprotein that mediates the phagocytosis of apoptotic cells. It shows ubiquitous pattern of expression in various types of cells and tissues. MFG-E8 is secreted from dendritic cells, including bone marrow-derived immature dendritic cells, follicular dendritic cells in the germinal center, and Langerhans cells in the skin [55, 56]. MFG-E8 is also expressed and secreted from bone marrow-derived macrophages and peritoneal macrophages after activation. It has been reported that bone marrow-derived immature dendritic cells secrete 30-fold more MFG-E8 than bone marrow-derived macrophages in the presence of granulocyte-macrophage colony-stimulating factor (GM-CSF) [55]. GM-CSF also increases the secretion of MFG-E8 from macrophages [26, 55], which is enhanced by fractalkine (CX3CL1) [25, 37].

Intensive immunohistochemical staining studies have revealed MFG-E8 expression and localization in various types of tissues. MFG-E8 is expressed around and on the apical surface of the alveolar epithelium of involuting mammary glands and the MFG-E8 secreted from the nascent cells was localized to the lumen and the apical surfaces of the epithelium [57]. Another immunostaining study of mouse and rat eyes showed that MFG-E8 is expressed in the inner layer of photoreceptor cells and in retinal pigment epithelial cells [58]. MFG-E8 expression has also been found in intestinal tissues. The abundant expression of MFG-E8 was observed in the cytoplasmic and extracellular regions of the lamina propria and the mononuclear cells of the injured colonic mucosa in the intestinal tissues of mice with acute colitis [20]. MFG-E8 has also been detected in skin tissue and is expressed in the spinous layer of murine skin and is also localized in the cells between the basal and surface layers of neoplastic skin tissue [59].

### 2.3. Regulation of MFG-E8 Expression

Since altered expression of MFG-E8 causes disruptions of homeostasis and is correlated with a number of diseases, the tight regulation of MFG-E8 expression is critical. A number of studies have identified various factors that modulate the expression of MFG-E8. Taking advantage of the function of MFG-E8 binding with the apoptotic cells to be phagocytosed and removed by macrophages, an* in vitro* study clearly showed that the coculture of macrophages with apoptotic cells highly induced MFG-E8 levels as compared to macrophage culture alone [29]. Some molecules of the innate immune system have been reported to regulate MFG-E8 expression. Fractalkine, also known as chemokine C-X3-C motif ligand 1 (CX3CL1) and neurotactin in mice, is primarily expressed in endothelial cells and has the function of chemoattracting immune cells and promoting the strong adhesion of leukocytes to activated endothelial cells. It has been shown that fractalkine induces MFG-E8 expression in microglial cells through its cellular receptor CX_3_CR1 [25]. Granulocyte monocyte-colony stimulating factor (GM-CSF), a white blood cell growth factor, is a cytokine that differentiates stem cells into granulocytes and protects against tumors and infection. Jinushi et al. reported that GM-CSF is required for MFG-E8 expression in antigen presenting cells (APCs) for MFG-E8-mediated uptake of apoptotic cells in GM-CSF-triggered immunity [26]. In addition, the expression of MFG-E8 in macrophages isolated from various organs in GM-CSF-deficient mice was dramatically decreased. MFG-E8 expression is upregulated during lactation when prolactin hormone production is high in the mammary glands and circulated in the blood [27, 60]. Based on this observation, an* in vitro* study was performed to determine whether prolactin could regulate MFG-E8 expression which clearly showed that prolactin upregulated MFG-E8 expression in mammary epithelial cells and macrophages via the prolactin receptor [27, 28]. Peroxisome proliferator-activated receptor-delta- (PPAR-) *δ*, a member of the nuclear receptor proteins, is a transcriptional sensor for apoptotic cells, and PRAP-*δ*-deficient mice have been shown to develop autoimmune diseases due to the accumulation of apoptotic cells in the body. It has been reported that PPAR-*δ* induces the expression of opsonin genes, including MFG-E8, to remove apoptotic cells and to maintain self-tolerance by macrophages [29]. MFG-E8 expression is also known to be downregulated by several factors. Lipopolysaccharide (LPS) is a component of the cell wall of gram negative bacteria that induces inflammatory responses in innate cells, such as macrophages. It was reported that the expression levels of MFG-E8 in the serum, spleen, and other organs of mice injected with LPS were significantly decreased [33, 34], and, in accordance with this observation, LPS downregulated MFG-E8 expression in mouse peritoneal macrophages and the macrophage-like cell line, Raw264.7, via toll-like receptor 4 (TLR4)/CD14 signaling pathways [34]. The expression of gap junction protein plays a critical role in controlling the growth of a variety of transformed cells, likely regulating the expression of a number of growth factors. The gap junction protein connexin 43 was reported to be a negative regulator of MFG-E8 expression and has been shown to inhibit the growth of glioma cells through the suppression of MFG-E8 expression [35].

Interestingly, MFG-E8 is differentially expressed and secreted in some disease conditions. The expression of MFG-E8 in humans and in some animal models is reduced in autoimmune disease [18], rheumatoid arthritis (RA) [36], sepsis [34, 37–40], acute colitis [20], atherosclerosis [41], ischemia/reperfusion (I/R) injury [42], and Alzheimer's disease [43]. In contrast, MFG-E8 expression is elevated in other disease conditions, such as lung fibrosis [22], melanoma [30], breast cancer [31], and systemic lupus erythematosus (SLE) [32]. The regulation of MFG-E8 expression is summarized in Table 1.

### 2.4. Mechanism of Action and Functions of MFG-E8

**Figure 2:**
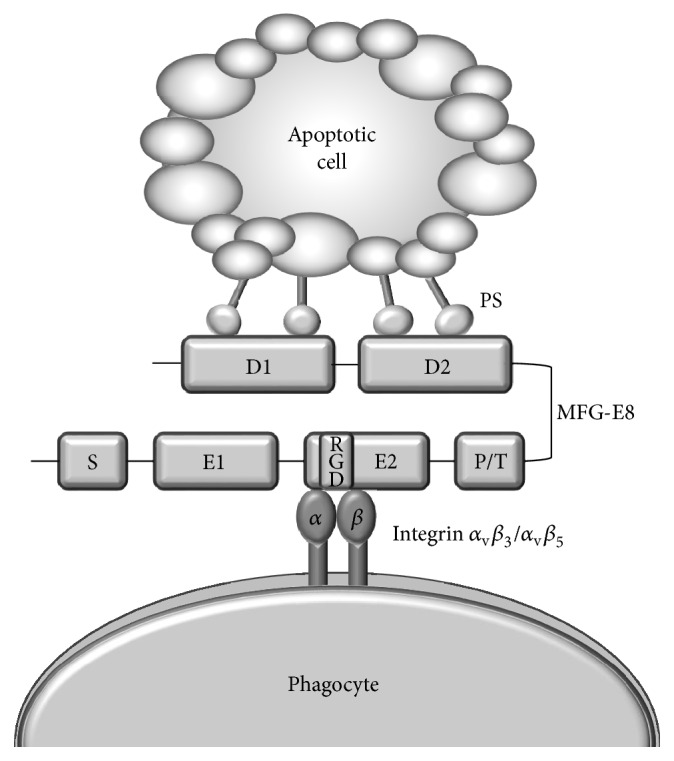
Mechanism of action of MFG-E8.

The most remarkable function of MFG-E8 is the phagocytosis and removal of apoptotic cells. The recognition of apoptotic cells is thought to be accomplished by the release of so-called “eat-me” signals from apoptotic cells, which recruit phagocytes such as macrophages and dendritic cells, leading to the clearance of dying cells [61, 62]. One of the major characteristics of apoptotic cells is to expose PS as an “eat-me” signal from its inner membrane leaflet to the external membrane surface, which is the key process for apoptotic cells to be recognized and engulfed by phagocytes. Although several receptors and soluble proteins that recognize PS on apoptotic cells have been reported, the mechanisms through which they clear apoptotic cells have been poorly understood. MFG-E8 was first reported to bind with PS and mediate the phagocytosis of apoptotic cells by peritoneal macrophages [19]. MFG-E8 produced from activated peritoneal macrophages binds with the PS of apoptotic cells through its C-terminal discoidin domains and also attaches to the *α*_v_ *β*_3_/*α*_v_ *β*_5_-integrin expressed on activated macrophages through the terminal RGD motif of the EGF repeats (Figure 2). The formation of this MFG-E8-mediated complex mediates conformational changes in integrin that triggers signal transduction, leading to cytoskeletal rearrangements in the macrophages that enhance the phagocytic capacity of apoptotic cells. The deletion of C-terminal discoidin domains results in a loss of the ability to bind with PS in the membrane of apoptotic cells [53], and a point mutation of the RGD motif in the EGF repeat inhibits phagocytosis through the masking of PS in apoptotic cell membranes [63]. There is evidence that the levels of MFG-E8 are critical for its function. The phagocytosis of apoptotic cells is enhanced by low levels of human MFG-E8 (hMFG-E8), whereas high levels of hMFG-E8 inhibit phagocytosis in a dose-dependent manner [32]. Similar to its role in the clearance of apoptotic cells, MFG-E8 also removes a variety of unwanted molecules, debris, and microvesicles from different organs. MFG-E8 has been reported to remove excessive collagen in lung tissues, binding with collagen through its two discoidin domains, and facilitating collagen uptake by alveolar macrophages [22]. MFG-E8 is also involved in the clearance of defective red blood cells (e.g., sickle cells), mediating phagocytosis by macrophages in the blood. This role suggests that MFG-E8 has an important function in protecting the body from diseases due to abnormal red blood cells, such as sickle cell anemia.

MFG-E8-deficiency results in the impairment of the ability to remove apoptotic cells and disrupts the homeostasis of bodily functions. New insights have been gained through the* in vivo* study of MFG-E8^−/−^ mice, which have been generated by deleting exons 4 to 66 of the MFG-E8 gene [18]. In wild-type mice, a large number of lymphocytes generated from the bone marrow form germinal centers in the spleen and lymph nodes. During germinal center formation, a massive number of cells undergo apoptosis, a process that is highly regulated. In MFG-E8^−/−^ mice, multiple enlarged germinal center and splenomegaly are observed, indicating that MFG-E8 is critical for the appropriate apoptosis of lymphocytes during the development of the immune organs. MFG-E8 is also critical for the phagocytosis of apoptotic epithelial cells in the mammary glands, which is important for its involution. MFG-E8^−/−^ mice were observed to have macrophages with an impaired ability to clear apoptotic epithelial cells due to the lack of MFG-E8 and the inefficient redevelopment of the mammary glands, leading to impairment of mammary gland involution [64]. Therefore, MFG-E8 plays a crucial role in the recognition and removal of apoptotic cells by phagocytosis, and its deficiency results in a breakdown in self-tolerance and homeostasis.

## 3. MFG-E8 in Inflammatory Responses and Inflammatory/Autoimmune Diseases

### 3.1. Role of MFG-E8 in LPS-TLR4 Signaling

**Figure 3:**
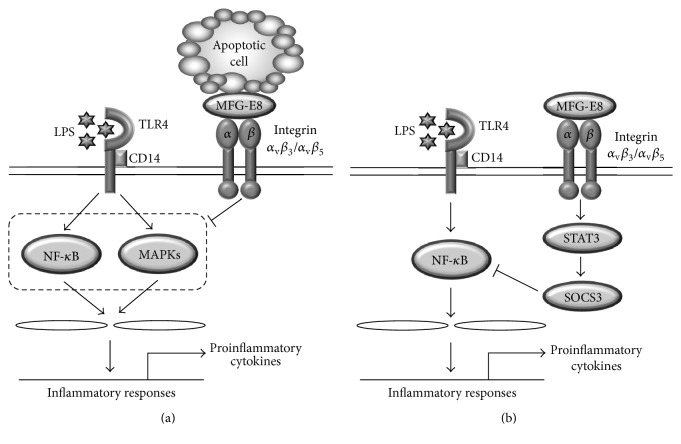
Anti-inflammatory mechanisms of MFG-E8 in a LPS-TLR4 signaling pathway. (a) Apoptotic cell-dependent anti-inflammatory role of MFG-E8. MFG-E8 mediates the phagocytosis of apoptotic cells, leading to the suppression of proinflammatory cytokine production through the inhibition of NF-*κ*B and MAPK signaling pathways. (b) Apoptotic cell-independent anti-inflammatory role of MFG-E8. MFG-E8 binds with integrin *α*_v_ *β*_3_/*α*_v_ *β*_5_ and induces STAT3-mediated SOCS3 activation, leading to the inhibition of the NF-*κ*B signaling pathway, thereby suppressing the production of proinflammatory cytokines.

TLR4 is the biological receptor for LPS, one of the most abundant and pathogenic molecules in the cell walls of gram negative bacteria. LPS-triggered TLR4 signaling activates inflammatory responses through the activation of nuclear factor kappa B (NF-*κ*B) and mitogen-activated protein kinase (MAK) signaling pathways, leading to the induction of proinflammatory cytokines such as tumor necrosis factor-alpha (TNF-*α*), interleukin-1 beta (IL-1*β*), and IL-6, as well as inflammatory mediators such as nitric oxide (NO), reactive oxygen/nitrogen species (ROS/RNS), and prostaglandin E_2_ (PGF_2_). It has been demonstrated that MFG-E8 expression is regulated by the LPS-TLR4 signaling pathway.* In vitro* and* ex vivo* studies clearly show that LPS downregulates the expression of MFG-E8 in both Raw264.7 murine macrophage cell lines and peritoneal macrophages [37, 39]. This observation was confirmed* in vivo* by cecal ligation and puncture (CLP) sepsis animal models. The splenic mRNA expression of MFG-E8 was reduced in the CLP sepsis mice injected with LPS in a dose-dependent manner, and this reduction was significantly attenuated by the administration of polymyxin B, which neutralizes LPS. In addition, this suppression of splenic mRNA expression of MFG-E8 in CLP sepsis animals was not observed in TLR4-mutated or CD14^−/−^ mice [34], strongly indicating that MFG-E8 expression is negatively regulated by LPS through the TLR4 signaling pathway. Moreover, MFG-E8 has also been reported to have an anti-inflammatory role in LPS-TLR4-mediated inflammatory responses during the phagocytosis of apoptotic cells. In coculture conditions of macrophages with apoptotic cells, MFG-E8 decreased the LPS-induced production of proinflammatory cytokines from macrophages through the suppression of the activities of intracellular signaling molecules, including p38, extracellular signal-regulated kinase (ERK)1/2, and c-Jun N-terminal kinase (JNK) in the mitogen-activated protein kinase (MAPK) signaling pathway as well as the activity of p65 in the NF-*κ*B signaling pathway [33]. In accordance with this observation, MFG-E8 suppressed inflammation in sepsis and ischemia/reperfusion conditions with LPS-TLR4 signaling by reducing the production of proinflammatory cytokines such as TNF-*α*, IL-1*β*, and IL-6 [21, 40]. Moreover, the uptake of apoptotic cells significantly suppressed LPS-induced inflammatory responses not only through decreasing the production of proinflammatory cytokines including TNF-*α* and IL-12, but also by increasing the production of the anti-inflammatory cytokine IL-10. This anti-inflammatory action was mediated by PPAR-d, which can induce the expression of MFG-E8 and other types of opsonins [29]. MFG-E8 also shows a direct anti-inflammatory role independent of the phagocytic engulfment of apoptotic cells. Recombinant MFG-E8 lowered intestinal inflammation by directly regulating TLR4 signaling through its binding with integrin *α*_v_ *β*_3_, and the phagocytosis of apoptotic cells was not involved in this MFG-E8-mediated suppression of inflammation [20]. Another study revealed the direct anti-inflammatory function of MFG-E8 through a different mechanism. MFG-E8 suppressed inflammatory responses and in turn the production of TNF-*α* in macrophages through the STAT-3-mediated activation of suppressor of cytokine signaling 3 (SOCS3), which is a negative regulator of the LPS-induced TLR4 signaling pathway by the inhibition of NF-*κ*B p65 [65]. These findings strongly support the anti-inflammatory role of MFG-E8 in the LPS-TLR4 signaling pathway, and this MFG-E8-mediated anti-inflammatory function is achieved through the phagocytosis of apoptotic cells as well as the direct inhibition of LPS-TLR4 signaling independent of the phagocytosis of apoptotic cells (Figure 3).

### 3.2. Role of MFG-E8 in Inflammatory/Autoimmune Diseases

#### 3.2.1. SLE

The expression level of MFG-E8 has been thought to correlate with the pathogenesis of SLE. MFG-E8^−/−^ mice showed many unengulfed apoptotic cells in the germinal centers of the spleen, and autoimmune disease was induced in these mice with symptoms similar to that of human SLE, indicating that MFG-E8 has a critical role in removing apoptotic cells and that the complete loss of MFG-E8 causes SLE-like autoimmune disease [18]. Interestingly, an* in vitro* study demonstrated that MFG-E8-mediated engulfment of apoptotic cells shows a bell-shaped efficiency curve in response to MFG-E8 at increasing concentrations in the macrophages isolated from MFG-E8 wild-type mice [32], indicating that the ability of macrophages to engulf apoptotic cells is also inhibited by high levels of MFG-E8. This could be explained by the bridge model for the engulfment of apoptotic cells by macrophages. High levels of MFG-E8 can saturate both apoptotic cells and macrophages, leading to the masking of the bridging between apoptotic cells and macrophages, thereby inhibiting the engulfment of apoptotic cells [32]. The correlation between MFG-E8 and SLE has also been studied in humans. The serum level of MFG-E8 was found to be higher in SLE patients as compared to very low or undetectable levels in healthy controls; in addition, the medical treatment of SLE resulted in significantly lower levels of MFG-E8 in patients [32]. Genetic variations in the MFG-E8 gene have also been studied in SLE patients. One study compared single nucleotide polymorphisms in the coding region of MFG-E8 between healthy controls and SLE patients and found that genetic polymorphisms on MFG-E8 residue 76(Met) allele predisposed participants to SLE in a recessive manner, while the variation of the MFG-E8 residue 76(Leu) allele was negatively associated with SLE [66].

#### 3.2.2. Rheumatoid Arthritis and Osteoarthritis

Some studies have demonstrated that MFG-E8 plays a role in inflammatory degenerative bone diseases such as RA and osteoarthritis (OA). The expression of MFG-E8 is downregulated in inflammatory conditions and has also been found to be downregulated in the sera of RA patients, while an* in vitro* study revealed that MFG-E8 suppresses inflammatory responses by suppressing the production of proinflammatory cytokines. Moreover, the expression of MFG-E8 is decreased in arthritic mice, and the loss of MFG-E8 exacerbated arthritis and led to more severe bone loss in mice by inducing the production of proinflammatory cytokines and the infiltration of pathogenic neutrophils in the inflamed joints [36]. MFG-E8 is also expressed in human and mouse osteoclasts and modulates their development and function. Genetic deletion of the MFG-E8 gene and antibody-mediated neutralization of MFG-E8 clearly induced osteoclastogenesis and enhances inflammation-induced bone loss, whereas local administration of MFG-E8 prevented bone loss in mice [67]. This observation was confirmed by another* in vivo* study showing that the loss of MFG-E8 in mice resulted in low bone mass and promoted ovariectomy-associated bone loss by inducing osteoclastogenesis [68].

#### 3.2.3. Sepsis

Sepsis is a high lethal systemic inflammatory disease characterized by the increase in proinflammatory cytokines and the accumulation of apoptotic cells. It has been reported that MFG-E8 is correlated with sepsis. During sepsis, a large number of immune cells undergo apoptosis due to the impairment of apoptotic cell clearance, which then induces secondary necrotic cell development that dysregulates proper immune function and induces the production of proinflammatory cytokines [69–71]. Therefore, studying the function of MFG-E8 could lead to promising therapeutic approaches that facilitate the clearance of apoptotic cells by MFG-E8 in sepsis. Miksa et al. induced a CLP sepsis model in rats and assessed the protein levels of MFG-E8 as well as thymocyte apoptosis. In these rats, the MFG-E8 protein levels were decreased in the spleen and liver by 48% and 70%, respectively, and thymocyte apoptosis was increased 1.6-fold. Moreover, inhibition of MFG-E8 in these rats completely abolished the apoptosis of thymocytes [39]. The regulation of MFG-E8 expression in sepsis was also confirmed by another study, which found that MFG-E8 protein levels in CLP rats were decreased in the spleen and the blood, where the clearance of apoptotic cells was impaired, and that the administration of immature dendritic cell- (IDC-) derived exosomes containing MFG-E8 facilitated the phagocytosis of apoptotic cells leading to a reduction in mortality [40]. It has been reported that MFG-E8 is constitutively expressed in the macrophages of the intestinal lamina propria in mice and that intestinal MFG-E8 expression was decreased in murine models of sepsis [38]. From these studies, it is clear that MFG-E8 expression is downregulated in sepsis, which contributes to the impairment in the clearance of apoptotic cells. The production of proinflammatory cytokines during sepsis is a critical cause of mortality and morbidity in sepsis animal models and in sepsis patients. MFG-E8 has been reported to negatively regulate the production of proinflammatory cytokines in inflammatory/autoimmune diseases and therefore could play a crucial role in sepsis by modulating the inflammatory response. The administration of IDC-derived exosome containing MFG-E8 significantly suppressed the expression of proinflammatory cytokines, including TNF-*α*, IL-6, and High Mobility Group Box 1 (HMGB1), in CLP sepsis animals [39, 40]. In addition, the administration of fractalkine in CLP sepsis animals suppressed the increase in blood lactate levels, liver injury, and proinflammatory mediators, which is associated with decreased MFG-E8 levels [37]. Several* in vivo* studies have demonstrated that MFG-E8 ameliorates the symptoms of sepsis in animal models. The administration of both MFG-E8-containing exosome and recombinant MFG-E8 significantly increased the survival rate by 80% in a CLP sepsis rat model [39, 40]. Moreover, the survival rate of sepsis was dramatically reduced in MFG-E8-deficient mice as compared to wild-type mice [40]. Although the role of MFG-E8 in sepsis has not been fully elucidated and requires further investigation, these studies strongly suggest that MFG-E8 plays a pivotal role in sepsis via the phagocytosis of apoptotic cells and the modulation of proinflammatory cytokine production, leading to anti-inflammatory effects and survival benefits.

#### 3.2.4. Ischemia/Reperfusion Injury

Ischemia/reperfusion (I/R) injury induces massive apoptotic cell death of the target organ as well as remote organs and also results in the inappropriate clearance of apoptotic cells [72–74]. I/R injury is another inflammatory/autoimmune disease characterized by the production of reactive oxygen species [75], cytokine secretion [76], complement activation [77, 78], mitochondrial dysregulation [79], activated leukocyte recruitment [80, 81], eicosanoid production [82, 83], and cell necrosis and apoptosis [84, 85]. An* in vivo* study using a mouse intestinal I/R injury model found that the expression levels of MFG-E8 mRNA and protein were significantly decreased in the spleen and lung by 50% and 60%, respectively, and that the administration of recombinant MFG-E8 in diseased mice dramatically improved the signs of I/R injury, including the increased levels of proinflammatory cytokines such as TNF-*α*, IL-1*β*, and IL-6, as well as histological markers of organ damage such as serum lactate levels, LDH, ALT, AST, and creatinine, resulting in an increase in the survival rate [42]. In addition, the administration of recombinant MFG-E8 decreased the accumulation of apoptotic cells in mouse lungs affected by I/R injury [42]. One recent* in vivo* study using a renal I/R mouse model further revealed that MFG-E8 expressed in macrophages and dendritic cells of the kidneys was reduced in the experimental mice, while the administration of recombinant MFG-E8 attenuated the signs of renal I/R injury, including renal function, histological tubular injury, and the induced levels of proinflammatory mediators such as IL-1*β*, IL-6, macrophage inflammatory protein-2, and myeloperoxidase [21]. These findings strongly indicate that MFG-E8 prevents organ damage under I/R conditions through its anti-inflammatory properties and could be an efficient therapeutic tool in I/R injury.

#### 3.2.5. Atherosclerosis

MFG-E8 is also expressed in both normal and atherosclerotic arteries in humans and has been reported to play a role in the phagocytic clearance of apoptotic cells by macrophages. The physiological role of MFG-E8 in the development of atherosclerosis was examined by reconstituting wild-type or* Mfg-e8*-deficient bone marrow into irradiated atherosclerosis-susceptible mice.* MFG-E8*-deficiency in a murine model of atherosclerosis resulted in the mice accumulating apoptotic debris due to decreased apoptotic cell phagocytosis, leading to the acceleration of atherosclerosis [41]. MFG-E8-deficiency also regulated the expression of cytokines and the activity of immune cells. MFG-E8-deficiency significantly downregulated the expression of the anti-inflammatory cytokine IL-10 in the spleen, while the expression of interferon-*γ* was increased in the spleen and arteries of the atherosclerotic mice reconstituted with* Mfg-e8*-deficient bone marrow [41]. Moreover, MFG-E8-deficiency reduced the suppressive functions of regulatory T-cells in the experimental mice [41]. This study suggests that the lack of MFG-E8 promotes the accumulation of apoptotic cells in atherosclerosis and suppresses the protective anti-inflammatory responses, thereby leading to acceleration of disease development.

## 4. MFG-E8-Targeted Drug Development

### 4.1. Inhibitors and Drugs Targeting MFG-E8

A number of studies have directly targeted MFG-E8 or indirectly intervened in MFG-E8 signaling in various diseases. A humanized monoclonal antibody specific for MFG-E8/lactadherin, Angiolix, showed potential for the treatment of breast and ovarian cancers [86]. Moreover, several studies have reported that the direct targeting of MFG-E8 suppresses the proliferation and invasion of vascular smooth muscle cells as well as the apoptosis of arterial endothelial cells [87–89]. Several studies have also found that MFG-E8 and its signaling are indirectly regulated by some natural products, drugs, and inhibitors. Polyphenol polymers, including resveratrol and the grape-seed proanthocyanidin extract procyanidin, which are found in many products, significantly reduced the expression of MFG-E8 induced by advanced glycation end products in arterial endothelial cells [90], leading to significant inhibition of apoptosis in arterial endothelial cells [88, 89]. Some antidisease drugs have also shown anti-MFG-E8 activity. Enalapril is an angiotensin converting enzyme inhibitor that is commonly used in the treatment of hypertension and heart diseases. Enalapril dramatically reduced MFG-E8 levels in the aorta of experimental hypertensive rats, with concomitant reductions in indices of arterial stiffness and the pulse wave velocity [91]. Although this study did not specify the type of cells in which MFG-E8 expression was decreased by Enalapril, it is clear that reduction of MFG-E8 ameliorates the symptoms of hypertension. *β*-receptor blockers, another agent used in hypertensive and heart disease, have also been shown to suppress MFG-E8 expression in vascular smooth muscle cells and help maintain vascular smooth muscle cell quiescence [92]. It has been reported that MFG-E8 production is increased by insulin and glucose in adipocytes [93], implying that MFG-E8 expression could be regulated by antidiabetic drugs. Glibenclamide is a drug used for the treatment of type 2 diabetes through the regulation of plasma glucose levels. It may be possible to use glibenclamide to target MFG-E8 expression and its substantial effects in inflammatory responses and inflammatory diseases. The potential of antidiabetic drugs to target MFG-E8, however, is unknown and molecular and cellular mechanism studies of MFG-E8 in diabetes need to be further investigated. Fluvastatin, a 3-hydroxy-3-methylglutaryl-coenzyme A (HMG-CoA) reductase inhibitor, is a drug used for the treatment of hyperlipidemia. It has been reported that circulating microparticles mediated by MFG-E8 are associated with endothelial dysfunction [94–97]. Interestingly, the levels of these microparticles released from the TNF-*α*-activated cultured human coronary artery endothelial cells are decreased by fluvastatin, leading to improved endothelial functions, suggesting a relationship between antihyperlipidemic inhibitors and the suppression of MFG-E8 signaling [98]. However, the studies regarding how MFG-E8 is associated with hyperlipidemia need to be further investigated in molecular and cellular levels.

### 4.2. MFG-E8 Targeting in Macrophage-Mediated Inflammatory/Autoimmune Diseases

As discussed in Section 3, a number of studies have successfully shown that MFG-E8 plays a crucial role in inflammatory responses and inflammatory/autoimmune diseases, suggesting that the specific targeting of MFG-E8, whether to inhibit or induce its expression, could be a potential strategy to treat and prevent these diseases. Sepsis is a life-threatening inflammatory/autoimmune disease that arises when the body's responses to pathogenic infection injure its own tissues and organs, leading to morbidity and mortality. Miksa et al. investigated the role of MFG-E8 using a sepsis animal model induced in rats by cecal ligation and puncture. MFG-E8 protein levels were decreased in these rats, and the injection of dendritic cell-derived MFG-E8-containing exosomes significantly attenuated the systemic inflammatory responses in these experimental models of sepsis [39]. Yang et al. also reported that short peptides derived from MFG-E8 and flanking its RGD motif alleviated the symptoms of organ injury in sepsis, as reflected by the decreased plasma levels of markers of organ injury such as aspartate aminotransferase and proinflammatory cytokines, including IL-6 and TNF-*α* [44]. These studies strongly suggest that the administration of MFG-E8 could be a potential therapeutic for the prevention and treatment of sepsis. The phagocytosis of viable neurons is critical for brain pathology and inhibition of this process is beneficial. MFG-E8 expression is upregulated in microglial cells, and deficiency in its expression suppresses the phagocytosis of neurons and brain atrophy after focal brain ischemia [49], indicating that targeting MFG-E8 could be beneficial for brain ischemic injuries. A Japanese group has reported the role of MFG-F8 in colitis using an experimental animal model. The intrarectal administration of recombinant MFG-E8 in trinitrobenzene sulfonic acid-induced colitic mice significantly suppressed colon shortening, body weight loss, and histological inflammation [45]. This group further confirmed the anticolitic action of MFG-E8 using butyric acid in a different experimental model of colitis. Butyric acid attenuated the symptoms of experimental colitis induced by the administration of dextran sodium sulfate in C57BL/6N mice and wild-type mice, but this attenuation of symptoms was not observed in MFG-E8^−/−^ mice [46]. These two studies strongly suggest that MFG-E8 is involved in the pathogenesis of colitis and that the administration of MFG-E8 could be a therapeutic approach in the treatment of intestinal inflammatory diseases, including colitis. MFG-E8 was reported to be a critical player in arterial wall inflammatory remodeling during aging, hypertension, diabetes mellitus, and atherosclerosis. The exogenous addition or endogenous overexpression of MFG-E8 promotes the proliferation of vascular smooth muscle cells, whereas the silencing of MFG-E8 or the inhibition of its receptor results in the reduced proliferative ability of these cells [47]. These findings suggest that MFG-E8 could be a potential therapeutic agent for the age-associated increase in the proliferation of aortic vascular smooth muscle cells during arterial wall inflammatory remodeling. The phagocytic clearance of apoptotic cells is a crucial process for the prevention of inflammation and autoimmunity. Glucocorticoids ameliorate the symptoms of SLE in mice by enhancing the clearance of apoptotic cells, and the inhibition of MFG-E8 expression via RNA interference or genetic knockout significantly suppressed the phagocytosis-enhancing effects of glucocorticoids in these SLE mice, indicating that the MFG-E8-dependent induction of apoptotic cell clearance is critical for the anti-inflammatory effect of glucocorticoids (Table 2) [48]. Atherosclerosis is an inflammatory vascular disease characterized by artery wall thickening as a result of the invasion and accumulation of inflammatory immune cells and is considered to be one of the major complications of type 2 diabetes. Unlike other inflammatory/autoimmune diseases described above, the serum level of MFG-E8 was found to be high in patients with type 2 diabetes mellitus and was positively correlated with carotid-femoral pulse wave velocity. The inhibition of MFG-E8 expression by RNA interference in artic endothelial cells significantly ameliorated atherosclerosis, but the exogenous administration of recombinant MFG-E8 exacerbated the symptoms of atherosclerosis in experimental diabetic mice through the intracellular signaling molecules ERK and monocyte chemoattractant protein 1 [50]. Although it is still required to investigate the molecular and cellular mechanisms by which MFG-E8 expression is upregulated in this disease condition, this study suggests that MFG-E8 plays an important role in atherosclerosis in patients with type 2 diabetes mellitus and that targeting MFG-E8 could be a therapeutic approach for the treatment of atherosclerosis in type 2 diabetes mellitus. Taken together, it is clear that although MFG-E8 plays different roles depending on the clinical scenario, the selective targeting of MFG-E8 could be a promising strategy for the prevention and treatment of inflammatory/autoimmune diseases.

## 5. Conclusions and Perspectives

Inflammation is an essential biological process that protects our body from invasion by pathogens. However, uncontrolled and chronic inflammation leads to inflammatory/autoimmune diseases. Macrophages are major effector immune cells that govern inflammatory responses through various mechanisms. There is increasing evidence that the insufficient clearance of apoptotic or damaged cells is one of the main etiologies of inflammatory/autoimmune diseases, and a number of studies have demonstrated not only that MFG-E8 plays a key role in macrophage-mediated inflammatory responses, but also that the targeted inhibition or administration of MFG-E8 can ameliorate the symptoms of inflammatory/autoimmune diseases. In spite of these promising studies supporting the possibility of MFG-E8 as a therapeutic target for the treatment of inflammatory/autoimmune diseases, few drugs have been developed for these diseases. Targeting MFG-E8 could be a novel and promising strategy for the prevention and treatment of macrophage-mediated inflammatory/autoimmune diseases.

## Figures and Tables

**Table 1:** Altered expression of MFG-E8 in human diseases.

Factors	Localization	References
Upregulation of MFG-E8		
Fractalkine (CX3CL1)	Microglial cell, macrophage	[25]
GM-CSF	Macrophage, antigen presenting cell	[26]
Prolactin	Mammary epithelial cell, macrophage	[27, 28]
PPAR-*δ*	Macrophage	[29]
Lung fibrosis	Lung	[22]
Melanoma	Skin	[30]
Breast cancer	Breast	[31]
SLE	Blood	[32]

Downregulation of MFG-E8		
LPS	Macrophage, spleen, and blood	[33, 34]
Connexin 43	Mammary epithelial cell	[35]
Rheumatoid arthritis	Joints	[36]
Sepsis	Macrophage, spleen, and blood	[34, 37–40]
Acute colitis	Intestine	[20]
Atherosclerosis	Cardiovascular endothelial cell	[41]
Ischemia/reperfusion injury	Gut, spleen	[42]
Alzheimer's disease	Brain	[43]
Autoimmune diseases	Spleen, lymph node, and kidney	[18]

**Table 2:** MFG-E8 targeting in macrophage-mediated inflammatory/autoimmune diseases.

Disease model	MFG-E8	References
Sepsis	Anti-inflammatory	[39, 44]
Colitis	Anti-inflammatory	[45, 46]
Artery wall inflammatory remodeling	Anti-inflammatory	[47]
SLE	Anti-inflammatory	[48]
Brain ischemia	Inflammatory	[49]
Atherosclerosis	Inflammatory	[50]
